# MicroRNA Polymorphisms and Environmental Smoke Exposure as Risk Factors for Oesophageal Squamous Cell Carcinoma

**DOI:** 10.1371/journal.pone.0078520

**Published:** 2013-10-21

**Authors:** Yabing Wang, Matjaz Vogelsang, Georgia Schäfer, Marco Matejcic, M. Iqbal Parker

**Affiliations:** 1 International Centre for Genetic Engineering and Biotechnology, Cape Town, South Africa Component, UCT Campus, Observatory, Cape Town, South Africa; 2 IIDMM and Division of Medical Biochemistry, UCT Faculty of Health Sciences, Cape Town, South Africa; 3 National Institute of Chemistry, Ljubljana, Slovenia; Sanjay Gandhi Medical Institute, India

## Abstract

MicroRNAs (miRNAs) and related polymorphisms have been implicated in the susceptibility to oesophageal squamous cell carcinoma (OSCC). In our study, three miRNA-related SNPs: rs6505162 A>C (pre-miRNA of miR-423), rs213210 A>G (3’UTR of miR-219-1) and rs7372209 C>T (5’UTR of miR-26a-1) were investigated in the Black and Mixed Ancestry population groups in South Africa. The potential cumulative effects of these SNPs, as well as gene-environment interactions were also analysed. In Blacks, rs6505162 A>C was associated with OSCC under dominant, additive and recessive models with odds ratios (ORs) 1.353, 1.404, and 2.858, respectively. This locus showed very strong interactions with smoke inhalation from burning wood or charcoal used for heating and cooking in very poorly ventilated areas (OR_(GE)_=7.855, P_(GE)_=9.17*10^-10^ in the Black group). Furthermore, the miR-423-3p level was 1.39 fold up-regulated in tumour tissues compared to the adjacent normal tissue (paired t-test *P* value 0.0087). SNP-SNP interaction between rs2132210 and rs7372209 was found in both Black and Mixed Ancestry subjects. The AA_rs213210_-CT_rs7372209_ genotype had a protective effect on OSCC risk (in the Black, OR=0.229, *P*=0.012; and the Mixed Ancestry groups, OR=0.230, *P*=0.00014). This study is the first to link SNPs in miR-423 together with environmental smoke exposure to risk for developing OSCC.

## Introduction

Oesophageal cancer is the eighth most common cancer type and the sixth most common cause of cancer death worldwide[1]. There are two main histological types of oesophageal cancer. Adenocarcinoma, which is more common in developed countries and is likely to occur in individuals with Barrett’s oesophagus, and squamous cell carcinoma (OSCC) which predominates in developing countries. Africa, China and central Asia have the highest incidence of OSCC in the world, and in the Black African population the incidence rate reaches 16.3 per 100.000 population[1].

Although the actual aetiology of OSCC remains unclear, the lifestyle habits including nutrition, tobacco smoking and alcohol consumption are considered to be major environmental risk factors for OSCC[2–6]. The use of solid fossil fuels (mainly wood, coal and biomass) for open fire cooking and home heating has also been associated with risk of developing OSCC and lung cancer [7,8].

Polymorphisms in the miRNA genes have recently been found to be associated with OSCC risk in Chinese and White Americans [9,10]. MicroRNAs (miRNAs) are considered to have a critical role in post-transcriptional regulation of gene expression, usually by binding to the 3'-untranslated region (3'UTR) of their target mRNAs. Some miRNAs are involved in tumorigenesis by acting as either oncogenes or tumour supressor genes[11–19]. Several single nucleotide polymorphisms (SNPs) in miRNA or pre-miRNA are associated with different types of cancer susceptibility[20–22]. miRNA-related SNPs can affect the miRNA functions via three different mechanisms: 1) by altering transcription of the gene, 2) by interfering with pri-miRNA and pre-miRNA processing and 3) by changing the miRNA–mRNA interaction affinity[23]. Despite recent progress in understanding the role of miRNA SNPs in the aetiology of cancer, many mechanisms are still largely unclear and remain to be elucidated.

In this case-control study, we analysed association between 3 SNPs (rs6505162, located in pre-miRNA of miR-423; rs213210, located in 3’UTR of miR-219-1; and rs7372209, located in 5’UTR of miR-26a-1) and oesophageal cancer risk in the Black and Mixed Ancestry population groups of South Africa. All of these polymorphisms have been reported to be associated with OSCC in the White American population [9,10] and are located in different regions of relevant miR genes. We further investigated whether gene-environment interactions also play a role in OSCC, and if so, whether these miRNA SNPs are implicated. To our knowledge this is the first study investigating the relationship between polymorphisms in the miRNAs and environmental risk factors such as the inhalation of smoke from the combustion of solid fossil fuels in oesophageal carcinogenesis.

## Materials and Methods

### Study population and group criteria

The study population consisted of Black and Mixed Ancestry subjects from South Africa. The Black subjects were mainly Xhosa-speakers from the Eastern or Western Cape of South Africa. The Mixed Ancestry subjects were from the Western Cape and are an admixed population with major ancestral components from the indigenous Khoisan, Bantu-speaking Africans, Europeans and Asians. Blood samples from OSCC cases were collected at Groote Schuur Hospital and Tygerberg Hospital, both located in Cape Town, South Africa. Controls were healthy individuals without any previous history of cancer and were randomly recruited from the same population groups and geographical area as the cases. In the Black ancestry group, 368 cases and 583 controls were recruited, whereas 197 cases and 420 controls were of Mixed Ancestry. All OSCC cases were histologically confirmed according to ICD-10 guidelines. DNA was extracted from frozen blood samples using standard protocols[24]. Demographic data was collected through interviews conducted by professional research nurses. The main information included ethnicity, gender, age, smoking and drinking habits, as well as cooking methods during the last 20 years (Table 1). Subjects with current or former smoking habits were classified as smokers. Alcohol consumers were defined as individuals who consumed more than 40 grams of alcohol per day. In analysing cooking habits, we distinguished between modern nonsolid fuels (gas and electricity), and traditional solid fuels (charcoal and wood). Written informed consent was obtained from all participants. This study was approved by the University of Cape Town/Groote Schuur Hospital Human Ethics Research Committee.

**Table 1:** Main characteristics of OSCC cases and controls.

	Black Ancestry Group			Mixed Ancestry Group		
	Control (N=583, %)	Case (N=368, %)	*P*	Control (N=420 , %)	Case (N=197, %)	*P*
Age, Mean±SD	56.48±15.11	59.75±10.61	***<0.001***	57.70±14.38	61.20±10.42	***0.002***
Gender, Male (%)	237(40.65)	183(49.73)	***0.007***	123(29.29)	132(67.01)	***<0.001***
Smoking (%)Yes	240(41.16)	226(61.41)	***<0.001***	264(62.86 )	185(93.91)	***<0.001***
Alcohol (yes/medium/high)	197(33.79)	130(35.33)	0.623	107(25.48 )	76(38.58)	0.924
cooking with solid fuel	156(26.76)	205(55.71)	***<0.001***	89(21.19 )	69(35.03)	***<0.001***

### Genotyping

Three different types of SNPs in miRNA genes, namely rs6505162 (located in pre-miRNA of miR-423), rs213210 (located in 3’UTR of miR-219-1), and rs7372209 (located in 5’UTR of miR-26a-1) were selected based on previous studies, where they were found to be associated with increased oesophageal cancer risk in White Americans[9]. Genotyping was performed using the TaqMan allele discrimination assay according to the manufacturer’s instructions (Applied Biosystems, Life Technologies Corporation, Carlsbad, California, US). Briefly, probes were labelled with either VIC or FAM to detect the different alleles. Reactions were carried out in 5 µl volumes using 384-well PCR-plates with each reaction containing 5ng of DNA. Amplification reaction and fluorescent measurement were carried out using Roche LightCycler 480 II instrument (Roche Applied Science, Indianapolis, US) and the software SP4 1.5.0 to assign genotypes. The reaction conditions were as follows: 1) pre-incubation: 95 °C for 5min, 2) amplification: 92 °C for 15sec, 60 °C for 70sec, 47 cycles,3)cooling at 40 °C for 1min. Genotyping of 10% of randomly selected samples were replicated and analysed to evaluate the assay reproducibility.

### miR-423-3p expression level

A total of 85 histopathologically confirmed OSCC biopsies together with corresponding adjacent normal tissue samples were collected at Groote Schuur Hospital and Tygerberg Hospital, Cape Town, South Africa between 2008 and 2011. 

Total RNA was extracted from the tissue samples using RNeasy Mini Kit (Qiagen, Hilden, Germany) according to manufacturer’s protocol. To investigate the miR-423-3p expression level in tissue samples, we used a method described by Balcells et al (24), where poly(A) tailing of the miRNAs is followed by reverse transcription with a tagged poly(T) primer. Briefly, the reaction mix (10 µl) consisting of 100 ng of total RNA, 1 µl of 10x poly(A) polymerase buffer, 0.1 mM of ATP, 1 µM of reverse transcription poly(T) primer (5'-CAGGTCCAGTTTTTTTTTTTTTTTAGC-3’), 0.1 mM of dNTP mix, 100 units of MuLV reverse transcriptase (New England Biolabs, Hertfordshire, United Kindom) and 1 unit of poly(A) polymerase (New England Biolabs) was incubated at 42 °C for 1 hour followed by enzyme inactivation at 95 °C for 5 minutes. Quantification of microRNA was performed by PCR using the following primers: miR-423-3p forward primer, 5’-AGCTCGGTCTGAGGCCCCT-3’; miR-423-3p reverse primer, 5’-AGGTCCAG(T)_15_ACTGA-3’;18s RNA forward primer, 5’-TTTCGCTCTGGTCCGTCTTG-3’; 18s RNA reverse primer, 5’-TTCGGAACTGAGGCCATGAT-3’. MiR-423-3p levels were normalized to 18sRNA levels using the 2^(-ΔCt)^ model. Expression data was analysed with GraphPad Prism (version 5.01).

### Statistics

The IBM SPSS 19.0 software (New York, United States) was used to analyse the genotyping data. Student’s t-test was used to examine the average age difference between cases and controls. Chi-square test was used to examine categorical variables such as gender, smoking and drinking status in cases and controls.

For each SNP, enter method logistic regression was performed to compute Odds Ratio (OR) and 95% confidence interval (CI) adjusting for age, gender, smoking, alcohol consumption status, and smoke inhalation by cooking on open fires in poorly ventilated areas. Three different genetic models were tested for each SNP, including dominant model (code 0 for homozygous wild type and 1 for heterozygous or homozygous variant), additive model (code 0 for homozygous wild type, 1 for heterozygous and 2 for homozygous variant), and recessive model (code 0 for homozygous wild type or heterozygous and 1 for homozygous variant). The additive genetic model assumes that there is a linear gradient in risk between 0, 1 and 2 genotypes. Allelic odds ratio and *P* values were calculated using SHEsis software [25] (online version: http://analysis2.bio-x.cn/myAnalysis.php). Stratification analysis for tobacco smoking and cooking habits was performed using the SPSS package. 

Unadjusted significant *P*-values were corrected for multiple tests under the number of hypotheses tested (six in each ethnic group), using the Benjamini-Hochberg (BH) method [26] to avoid the False Discovery Rate (FDR). 

SNP-SNP interactions were first explored using the model-based multifactor dimensionality reduction (MB-MDR) approach by applying ‘mbmdr’ R-package to our whole dataset, as described by Calle et al [27].

In our study, multi-order interaction with the most significant association between a specific multi-locus genotype and the phenotype, was considered the best model and was further adjusted for other confounders using SPSS and corrected for multiple testing by 1000 permutations approach (P_1000_).

## Results

### Population characteristics

A total of 1565 individuals (565 OSCC cases and 1000 healthy controls) from African Black Ancestry group and Mixed Ancestry group were included in this study (Table 1). Among Black Ancestry cases, smokers were dominant (*P* < 0.001) and were more likely to inhale smoke from combustion of solid fossil fuels used for cooking and heating (*P*< 0.001) compared to controls. No significant difference was observed for alcohol consumption between cases and controls in both Black (P = 0.623) and Mixed Ancestry subjects (P = 0.924).

### Individual SNP Analysis and Cumulative Effect for OSCC Risk

We investigated associations of 3 individual microRNA SNPs with the risk of developing OSCC. Genotyping of the three SNPs was successful for 97% of samples, and all allele-genotype frequency distributions in controls were in Hardy-Weinberg equilibrium (*P* >0.05). The results in Table 2 show that SNPs rs6505162 and rs7372209 significantly altered the OSCC risk in South Africans. For the polymorphism in the pre-miRNA region of *miR-423* gene, the minor C allele occurred with a frequency of 22.8% in cases vs. 18.2% in control individuals in Black Ancestry subjects (*P* = 0.016). Furthermore, rs6505162 was positively associated with OSCC risk in an additive genetic model (adjusted OR = 1.404; *P* = 0.012) as well as in recessive model (adjusted OR = 2.858; *P* = 0.013) for the minor C allele in the Black population. Association remained significant after correction for multiple testing (in additive genetic model, *P*_corr_=0.036; in recessive model *P*_corr_ = 0.039). The rs7372209 T-allele in 5’UTR region of *mir26a-1* had a frequency of 13.8% in cases and 7.3% in healthy controls in the Mixed Ancestry group (*P* = 0.0094). Genotypic analysis showed a significantly reduced disease risk with adjusted ORs of 0.469 (in an additive genetic model for minor allele; *P* = 0.003) and 0.439 (in a dominant genetic model for minor allele; *P* = 0.002). Observed associations remained significant after correcting the *P*-values for mutliple testing (*P*_corr_ additive = 0.0105 and *P*_corr_ dominant = 0.014). Moreover, reduced cancer risk for *mir26a-1* rs7372209 (TT or CT versus CC) was also observed in the Black Ancestry subjects, with adjusted OR of 0.432 (*P* = 0.047). However, correction for multiple tests revealed borderline significance (*P* = 0.058).

**Table 2:** Polymorphisms in miRNAs and OSCC risk.

SNP (Genes)		Alleles					Genotypes			Adjusted OR^3^(95% CI) and *P*		
			Control Number (frequency)	CaseNumber (frequency)	OR^2^ (95% CI)	*P*		Control Number (frequency)	Case Number (frequency)	dominant	additive	recessive
rs6505162	Black	A^1^	936(0.818)	542(0.772)	1.328	***0.016***	AA	376 (0.66)	207(0.59)	1.353	1.404	2.858
(pre-miR-423)		C	208(0.182)	160(0.228)	(1.054-1.675)		AC	184(0.32)	128(0.37)	(1.000-1.831)	(1.077-1.830)	(1.242-6.574)
							CC	12(0.02)	16(0.05)	*P=0.050*	***P=0.012***	***P=0.013***
	Mixed	A^1^	584(0.695)	262(0.701)	0.975	0.853	AA	198(0.471)	89(0.476)	1.021	1.045	1.192
	Anc.	C	256(0.305)	112(0.299)	(0.748-1.272 )		AC	188(0.448)	84(0.449)	0.688-1.516	(0.760-1.437)	0.560-2.538
							CC	34(0.081)	14(0.075)	*P*=0.918	*P*=0.786	*P*=0.649
rs213210	Black	A^1^	1053(0.908)	661(0.918)	0.878	0.444	AA	473(0.816)	301(0.836)		0.818	
(3’UTR of miR-219-1)		G	107(0.092)	59(0.082)	(0.630-1.225)		AG	107(0.184)	59(0.164)	/^4^	(0.555-1.204)	/^4^
							GG	0	0		*P*=0.309	
	Mixed	A^1^	730(0.871)	344(0.896)	0.786	0.219	AA	311(0.742)	152(0.792)		0.690	
	Anc.	G	108(0.129)	40(0.104)	(0.535-1.155)		AG	108(0.258)	40(0.208)	/^4^	0.430-1.107	/^4^
							GG	0	0		*P*=0.124	
rs7372209	Black	C^1^	1124(0.972)	712(0.983)	0.592	0.121	CC	546(0.945)	350(0.967)		0.432	
(5’UTR of miR-26A-1)		T	32(0.028)	12(0.017)	(0.303-1.157)		CT	32(0.055)	12(0.033)	/^4^	(0.188-0.990)	/^4^
							TT	0	0		***P=0.047***	
	Mixed	C^1^	724(0.862)	358(0.927)	0.488	***0.0094***	CC	307(0.731)	166(0.860)	0.439	0.469	1.518
	Anc.	T	116(0.138)	28(0.073)	(0.317-0.752)		CT	110(0.262)	26(0.135)	(0.261-0.7380	(0.282-0.778)	(0.140-16.449)
							TT	3(0.007)	1(0.005)	P=0.002	***P=0.003***	P=0.731

1 Major alleles in each group.

2 Allelic Odds Ratio based on minor allele, regarding major allele as reference allele.

3 Adjusted for age, gender, tobacco smoking and cooking habits.

4 Not determined (no genotypes were found in one genotype group); can be considered as additive model.

We further evaluated possible joint effects of miRNA polymorphisms on oesophageal cancer risk. The potential interactions among the miRNA SNPs were first analysed with model-based multifactor dimensionality reduction (MD-MDR) analysis to find the best multi-locus interaction model. ORs for best interaction were further calculated and adjusted for other confounders. Cumulative effect of *miR*-*219-1* rs213210 and *miR-26a-1* rs7372209 (AA/CT genotype) was significantly associated with reduced cancer risk in the Black and Mixed Ancestry groups with ORs 0.229 (*P* = 0.012) and 0.230 (*P* = 0.0001), respectively (Table 3). After 1000 random permutations test interaction remained significant in the Mixed Ancestry subjects (P_1000_< 0.001) and borderline significant in the Black group (P_1000_ = 0.059).

**Table 3:** Cumulative effect of miRNA-related SNPs on OSCC risk.

	rs213210- rs7372209	control	case	OR adjusted^1^	*P* adjusted
Black	AA-CC	447(0.776)	292(0.260)	1(ref)	
	AA-CT	23(0.040)	5(0.014)	0.229(0.073-0.719)	***0.012***
	AG-CC	98(0.170)	51(0.144)	0.753(0.502-1.128)	0.169
	AG-CT	8(0.014)	6(0.017)	1.075(0.272-4.257)	0.918
Mixed Ancestry	AA-CC	243(0.579)	138(0.734)	1(ref)	
	AA-CT	69(0.164)	11(0.059)	0.230(0.108-0.491)	***0.00014***
	AG-CC	64(0.152)	24(0.128)	0.505(0.278-0.918)	***0.025***
	AG-CT	44(0.105)	15(0.080)	0.651(0.321-1.321)	0.234

1 Adjusted for age, gender, tobacco smoking and cooking habits

### SNP rs6505162 interaction with environmental smoke inhalation

Due to extremely low minor allele frequency in rs7372209, only rs6505162 was subjected to further gene-environment analysis. The potential interactions between the SNP rs6505162 and environmental exposures (e.g. tobacco smoking and cooking with solid fuel) were also analysed. No interaction was observed between rs6505162 and first-hand tobacco smoke exposure in association with OSCC risk (Table 4). While a 1.6-fold increase in OSCC risk was observed for *pre-miR-423* rs6505162 (AC/CC vs AA) in non-smokers, the observed 3.04 and 3.57-fold increased disease risk among rs6505162-AA and rs6505162-AC/CC smokers, compared to reference rs6505162-AA non-smokers, respectively, is thus solely the result of smoking. Similar effects were observed in the Mixed Ancestry group, where the cancer risk among rs6505162-AC/CC and rs6505162-AA smokers compared to reference rs6505162-AA non-smokers was 4.72 and 5.18-fold, respectively. 

**Table 4:** Interaction between SNP rs6505162 and smoking in OSCC risk.

	smoking	rs6505162	Control (freq)	Case (freq)	OR(95%CI) unadjusted	*P* unadjusted	OR adjusted^1^	*P* adjusted
Black	-	AA	219 (0.386)	79 (0.226)	1(ref)		1(ref)	
	-	AC+CC	113 (0.199)	57 (0.163)	1.398(0.929-2.106)	0.108	1.602(1.026-2.501)	***0.038***
	+	AA	153 (0.269)	127 (0.363)	2.301(1.624-3.261)	***2.79E-06***	3.040(1.992-4.640)	***2.56E-07***
	+	AC+CC	83 (0.146)	87 (0.249)	2.906(1.956-4.316)	***1.27E-07***	3.566(2.235-5.689	***9.57E-08***
Mixed Ancestry	-	AA	69 (0.166)	7 (0.038)	1(ref)		1(ref)	
	-	AC+CC	84 (0.202)	3 (0.016)	0.411(0.099-1.703)	0.22	0.447(0.106-1.888)	0.273
	+	AA	127 (0.305)	82 (0.441)	7.425(3.082-17.887)	***7.85E-06***	4.716(1.903-11.687)	***0.001***
	+	AC+CC	136 (0.327)	94 (0.505)	7.949(3.315-19.061)	***3.40E-06***	5.183(2.100-12.790)	***3.57E-04***

1 adjusted for age, gender, cooking habits.

When evaluating interaction between rs6505162 and usage of solid fuels for cooking, a strong interaction effect conferring OSCC risk was observed in the Black group (Table 5). Interaction between rs6505162 and solid fuel usage was associated with increased cancer risk with adjusted ORs of 1.75 (for rs6505162AC/CC carrier cooking with gas/electricity), 5.31 (for rs6505162AA carrier using solid fuels for cooking) and 7.86 (for rs6505162AC/CC carriers using solid fuels for cooking) in relation to reference rs6505162AA carriers cooking with gas/electricity. No interaction effect was observed in the Mixed Ancestry population.

**Table 5:** SNP rs6505162 interact with solid fuel cooking.

	Solid fuel use	SNP rs6505162	Ctrl	Case	OR(95%CI) unadjusted	*P* unadjusted	OR adjusted^1^	*P* adjusted
Black	-	AA	293 (0.529)	99 (0.286)	1(ref)		1(ref)	
	-	AC+CC	178 (0.321)	99 (0.286)	1.646(1.177-2.301)	***0.004***	1.746(1.238-2.464)	***0.001***
	+	AA	69 (0.125)	104 (0.301)	4.461(3.050-6.524)	***1.27E-14***	5.310(3.562-7.916)	***2.53E-16***
	+	AC+CC	14 (0.025)	44 (0.127)	9.302(4.889-17.695)	***1.07E-11***	7.855(4.061-15.194)^b^	***9.17E-10***
Mixed Ancestry	-	AA	58 (0.324)	117 (0.397)	1(ref)		1(ref)	
	-	AC+CC	73 (0.408)	134 (0.454)	1.099(0.719-1.680)	0.663	1.148(0.707-1.865)	0.576
	+	AA	27 (0.151)	25 (0.085)	2.179(1.162-4.084)	***0.015***	1.339(0.661-2.714)	0.418
	+	AC+CC	21 (0.117)	19 (0.064)	2.230(1.112-4.471)	***0.024***	1.390(0.621-3.114)	0.423

1 adjusted for age, gender, tobacco smoking.

### miR-423-3p expression is up-regulated in tumour tissue

**Figure 1:**
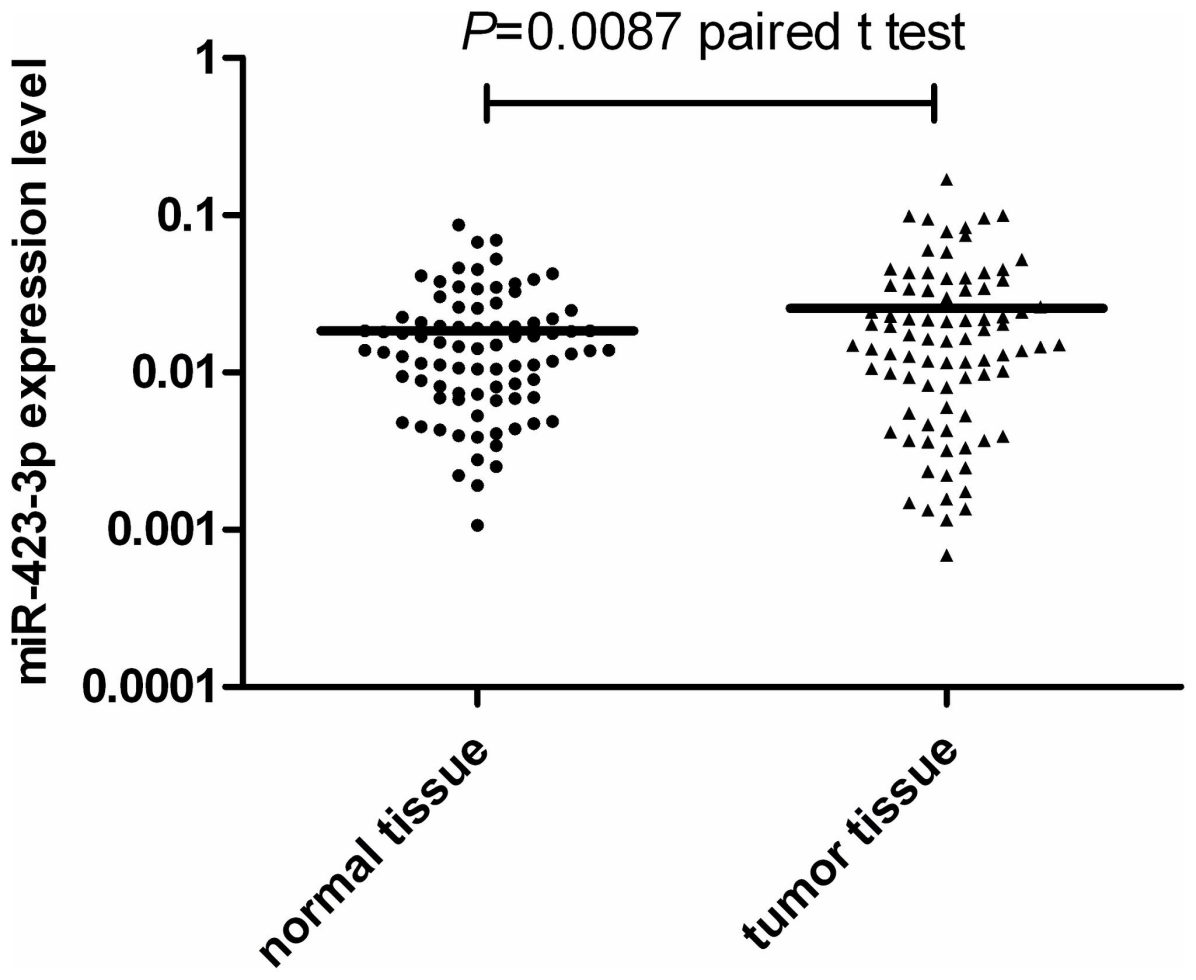
miR-423-3p expression is up regulated in tumour tissue. miR-423-3p levels were determined in normal and tumour tissue samples from OSCC patients (N=85) by RT-PCR as described in Materials and Methods (bars show group mean values). All values were normalized to 18s RNA levels. The Paired t-test was used to evaluate differences in expression levels between normal and tumour samples. Mean expression levels were: 1.843*10-2 in normal tissue samples and 2.562*10-2 in tumour tissue samples.

To further validate our results we investigated the levels of miR-423-3p in OSCC biopsies compared to corresponding normal tissue. We used the method of Balcells et al [28] to investigate the miR-423-3p levels in tumour and matched normal biopsy samples from OSCC patients. miR-423-3p was 1.39 fold over expressed in tumour compared to normal biopsies (mean_normal_ =1.843*10^-2^, mean_tumour_ =2.562*10^-2^, *P* = 0.0087; Figure 1). 

**Figure 2:**
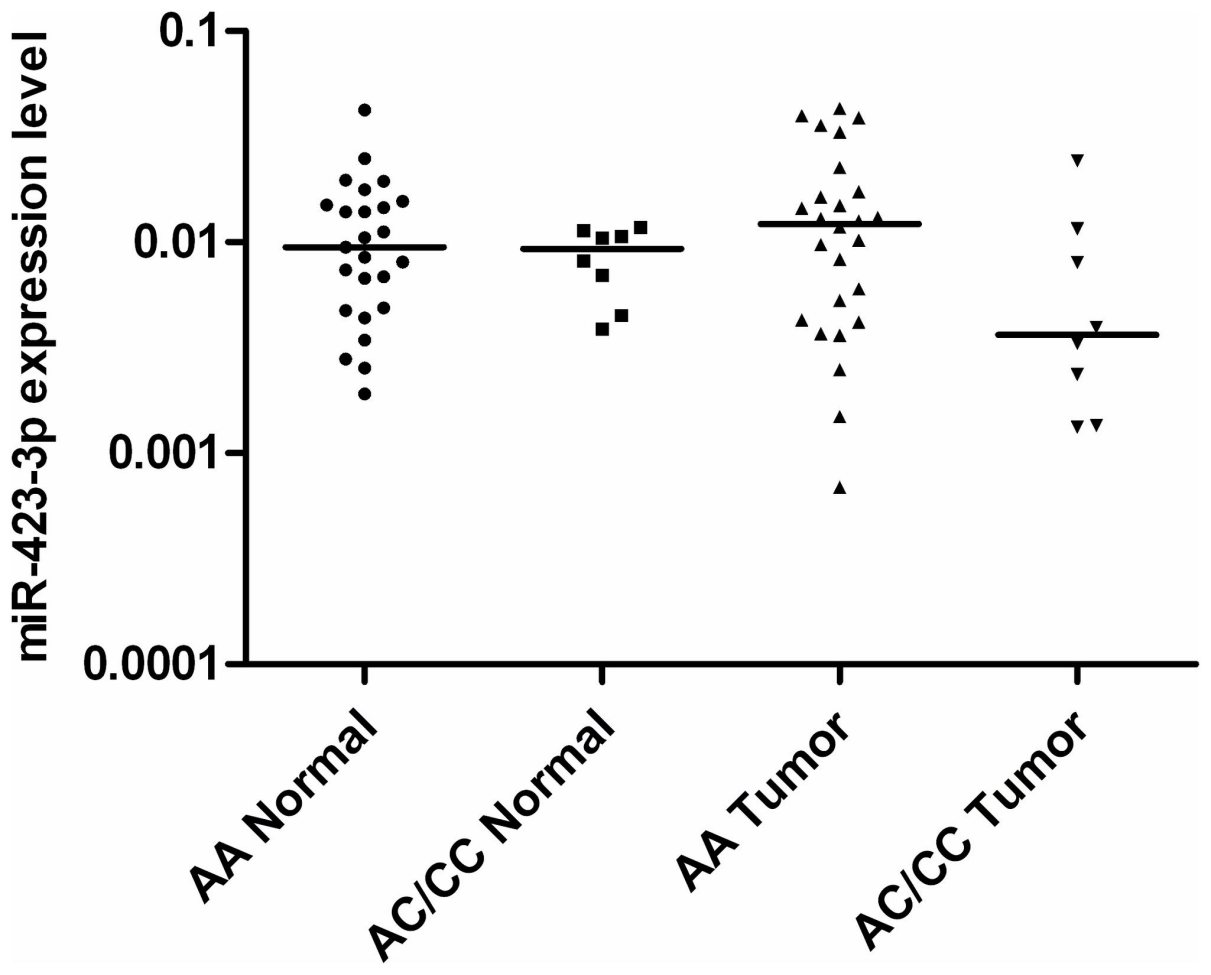
Expression of miR-423-3p does not correlate with rs6505162 genotypes. miR-423-3p levels were determined for different genotypes in normal and tumour tissue samples from OSCC patients (N = 32), respectively (bars show group mean values). All values were normalized to 18s RNA levels. Kruskal–Wallis one-way analysis of variance was used to evaluate differences in expression levels between groups. Mean values for rs6505162 genotypes are: 1.162*10-2 (AA, normal), 8.489*10-3 (AC/CC, normal), 1.487*10-2 (AA, tumour), and 7.029*10-3 (AC/CC, tumour).

To investigate whether SNP rs6505162 contributes to miR-423-3p over expression, we genotyped rs6505162 from blood DNA and compared the miR-423-3p expression between AA and AC/CC carriers in tumour and normal tissue samples, respectively. AC and CC carriers were combined due to extremely low frequency of CC genotype among patients. Results did not show any significant correlation between genotype and miR-423-3p expression in tumour or in normal biopsy sample (Figure 2).

## Discussion

Polymorphisms in miRNA genes have been associated with numerous cancer types including OSCC [29–32]. The underlying mechanism of such carcinogenesis may act through affecting the binding efficiency of miRNA to its target mRNAs. In the present study, we chose 3 miR SNPs that have previously been shown to be associated with oesophageal cancer [9]. Similar to the diverse distribution of gene related SNPs in different populations, the frequency of miRNA SNP also varies considerably among different populations [33]. In the rs213210 polymorphism, the homozygous genotype for the minor G allele was not observed among either Black or Mixed Ancestry subjects, however carriers have previously been observed among White Americans [9]. Similarly, we did not observe the homozygous genotype for the rs7372209 minor T allele in Black subjects. When compared to HapMap (Release 28; http://hapmap.ncbi.nlm.nih.gov/), the rs213210GG genotype frequency was 0.018 in the CEU, 0.006 in MKK (Kenya), 0.007 in YRI (Nigeria), and 0 in ASW (African ancestry in Southwest USA) populations, whereas in rs7372209, frequencies of homozygous genotype for minor T allele were 0.071 in CEU, 0.006 in MKK, and 0 in YRI and ASW population. The low frequencies of homozygous genotypes for minor alleles in the analysed SNPs in the HapMap populations of African origin confirm the reliability of our genotyping assay.

This study shows for the first time that the rs6505162 A>C SNP (miRNA-423) in the Black African population is associated with OSCC. The results are consistent with a previous study performed in the White American population, where the C allele confers increased risk for oesophageal cancer [9]. miRNA genes are transcribed as long primary transcripts (pri-miRNAs) that are subsequently cleaved by Drosha into shorter hairpin-shaped precursor miRNAs that are then exported to the cytoplasm, where they are further processed into ~22 bp mature miRNAs by Dicer. Since the rs6505162 polymorphismis located in the precusor of miR-423, it was reasonable to investigate whether rs6505162 affects miR-423-3p expression. Our results showed miRNA-423-3p is up-regulated in tumor tissues compared to adjacent normal tissue, which is consistent with a previous report, in hepatocellular carcinoma patients [32]. According to our results, expression of miR-423-3p does not correlate with the rs6505162 polymorphism, suggesting there must be other mechanisms by which rs6505162 confers elevated OSCC risk. The rs6505162 SNP is located in the region where it could affect three different genes: miR-423, miR-3184 and the nuclear speckle spicing regulatory protein 1 (NSRP1). Genetic regions of miR-423 and miR-3184 are overlapping at the position of rs6505162, and the two genes are oppositely orientated, situating the rs6505162 downstream of miR-423 and upstream of miR-3184. It is thus possible that rs6505162 modulates miR-3184 rather than miR-423.

As far as SNP rs7372209 is concerned, the T allele of rs7372209 (mir-26a-1) is associated with a 64% decreased risk for bladder cancer in females[34] and a two fold increased risk for premalignant oral lesions[35]. Our study further confirms the beneficial effect of rs7372209 in cancer development. In contrast to our results on squamous cell carcinoma, the T allele of rs7372209 was found to increase the risk of oesophageal adenocarcinoma in White Americans [9]. Our data suggest that there is an interaction between rs213210 (miR219-1) and rs7372209 (miR26a-1) in confering oesophageal cancer risk, with genotype AA_rs213210_-CT_rs7372209_ reducing the risk of cancer development in both the Black and Mixed Ancestry groups, respectively.

When interactions between genetic variants and environment were taken into account, the rs6505162 interacted with environmental smoke inhalation in Black Africans. Subjects with at least one rs6505162 C-allele and using solid fuels (e.g. wood and charcoal) for cooking had increased risk of developing OSCC compared to rs6505162 AA-genotype carriers that used electricity or gas for cooking. This effect was not observed in the Mixed Ancestry group, probably due to the population heterogeneity. The mixed population was formed about 300 years ago from the union of several different ethnic groups, receiving genetic contributions from the indigenous Khoi and San people and from Asian, European and sub-Saharan African populations [36]. No interaction was observed between rs6505162 and first-hand tobacco smoking in association with OSCC risk.

To our knowledge this is the first study investigating the joint effects between miRNA-related SNPs and environmental exposures in association with OSSC risk. Increasing evidence suggests that miRNAs help to confer robustness to biological processes by reinforcing transcriptional programs and attenuating aberrant transcription [37]. Our study supports the notion, that miRNA-related variants in combination with exposure to environmental risk factors contribute to OSCC, therefore confirming previous findings that interactions between genes and environment contribute to OSCC [3,38,39].
